# A Longitudinal Analysis of Mosquito Net Ownership and Use in an Indigenous Batwa Population after a Targeted Distribution

**DOI:** 10.1371/journal.pone.0154808

**Published:** 2016-05-04

**Authors:** Sierra Clark, Lea Berrang-Ford, Shuaib Lwasa, Didacus Namanya, Sabastian Twesigomwe, IHACC Research Team, Manisha Kulkarni

**Affiliations:** 1 Dept of Epidemiology, McGill University, Montreal, Quebec, Canada; 2 Dept of Geography, McGill University, Montreal, Quebec, Canada; 3 Dept of Geography, Makerere University, Kampala, Uganda; 4 Ministry of Health, Kampala, Uganda; 5 Batwa Development Programme, Buhoma, southwestern Uganda; 6 Indigenous Health Adaptation to Climate Change Research Team, McGill University, Montreal, Canada; 7 School of Epidemiology, Public Health and Preventive Medicine, University of Ottawa, Ottawa, Ontario, Canada; Tulane University School of Public Health, UNITED STATES

## Abstract

Major efforts for malaria prevention programs have gone into scaling up ownership and use of insecticidal mosquito nets, particularly in sub-Saharan Africa where the malaria burden is high. Socioeconomic inequities in access to long lasting insecticidal nets (LLINs) are reduced with free distributions of nets. However, the relationship between social factors and retention of nets after a free distribution has been less studied, particularly using a longitudinal approach. Our research aimed to estimate the ownership and use of LLINs, and examine the determinants of LLIN retention, within an Indigenous Batwa population after a free LLIN distribution. Two LLINs were given free of charge to each Batwa household in Kanungu District, Uganda in November 2012. Surveyors collected data on LLIN ownership and use through six cross-sectional surveys pre- and post-distribution. Household retention, within household access, and individual use of LLINs were assessed over an 18-month period. Socioeconomic determinants of household retention of LLINs post-distribution were modelled longitudinally using logistic regression with random effects. Direct house-to-house distribution of free LLINs did not result in sustainable increases in the ownership and use of LLINs. Three months post-distribution, only 73% of households owned at least one LLIN and this period also saw the greatest reduction in ownership compared to other study periods. Eighteen-months post distribution, only a third of households still owned a LLIN. Self-reported age-specific use of LLINs was generally higher for children under five, declined for children aged 6–12, and was highest for older adults aged over 35. In the model, household wealth was a significant predictor of LLIN retention, controlling for time and other variables. This research highlights on-going socioeconomic inequities in access to malaria prevention measures among the Batwa in southwestern Uganda, even after free distribution of LLINs, and provides critical information to inform local malaria programs on possible intervention entry-points to increase access and use among this marginalized population.

## Introduction

Insecticide-treated nets (ITNs) and long-lasting insecticidal nets (LLINs) are well recognized as cost-efficient and highly effective interventions for reducing the burden of malaria in endemic regions in sub-Saharan Africa [1]. Treating mosquito nets with an insecticide provides double protection: nets provide a direct barrier against host-biting mosquitoes for the person(s) sleeping under them; and mosquitoes may be killed if they come into contact with the insecticide, extending protection to non-users through reduced vector abundance [2]. Building on previous global targets, the recent World Health Organization (WHO) technical plan calls for a scale up of the distribution of LLINs to achieve universal coverage in high-risk areas in order to reduce malaria mortality by 40% of the current rate by 2020 and 90% by 2030 [3,4].

A core focus of free mosquito net distribution campaigns has been equitable coverage–within and between populations–with the goal of reducing disparities in access to preventive measures for economically or socially marginalized sub-populations. Previous studies show that large-scale, free net distribution campaigns can reduce inequities in household net ownership across socio-economic gradients, relative to other forms of distribution [5–10]. Inequities in ownership and use may still persist, however, preventing those among the lowest socioeconomic strata from accessing and retaining malaria preventative measures [10,11]. Socioeconomic factors, such as household wealth and education, have been identified as consistent and important predictors of mosquito net acquisition in the absence of free-distribution campaigns [7,8,12]. However, very few studies have longitudinally investigated the extent to which net retention and continued use are patterned by socio-economic gradients after a free distribution [11,13–16]. It has been postulated that, over the long-term, households that receive nets free of charge may experience greater net attrition compared to households that purchase nets. For example, in a study that investigated alternate uses of mosquito nets in Ghana, Senegal, Nigeria and Uganda, nets from a free distribution campaign were six-times more likely to be given away than nets purchased or obtained through other means [13]. Marginalized populations may be at a greater risk of net attrition following a free-distribution event, relative to less marginalized populations, if a lack of resources incentivizes an alternative use of the net [17–19]. Herein, our understanding of the role that socioeconomic gradients play in influencing the retention of LLINs by households remains limited.

Understanding patterns of LLIN ownership and use can provide contextual insight into possible entry points for interventions, including those targeted at increasing rates of LLIN access, LLIN hanging, and sleeping under existing LLINs [20]. According to a framework proposed by Vanden Eng *et al* [20], individuals can be classified into one of four categories of net use or non-use: 1) they may live in a household that does not own a net (*non-use*); 2) they may live in a household that owns a net, but does not hang the net (*non-use*); 3) they may live in a household that owns and hangs a net but they themselves do not sleep under it (*non-use*); or 4) they may sleep under a net *(use*). There is negligible research on access to, and use of, LLINs among Indigenous populations in sub-Saharan Africa, despite evidence of significant socioeconomic and health inequalities of these populations [21]. Estimation and characterization of LLIN non-use for high-risk sub-populations, particularly vulnerable and marginalized peoples, will be critical to meeting the Global Malaria Action Plan’s goals of universal coverage.

In Uganda, the Ministry of Health works in partnership with aid agencies to finance and strategize malaria control initiatives in high-risk regions and for high-risk individuals [22]. From the most recent Uganda Malaria Indicator Survey (MIS 2014), nearly 90% of all households in Uganda had at least one LLIN, 74% of children under five slept under a LLIN the night before the survey, and it was estimated that 79% of the population had access to an ITN [22]. The Uganda MIS 2014 reported that amongst the poorest households, a higher proportion owned at least one LLIN (91.5%) compared to amongst the wealthiest households (84.5%), which is likely the result of mass net distribution campaigns over the past decade. Indeed, 93.3% of nets owned by the poorest households were obtained from a campaign compared to 68% among the wealthiest households. Furthermore, only 2.1% of nets owned by the poorest households were purchased at a pharmacy, shop, or market, compared to 24.6% of nets obtained by households in the wealthiest quintile [22]. Within the context of mass distribution campaigns, even though household socioeconomic gradients do not appear to disadvantage net ownership for the poorest households at the national level, the most socially and ethnically marginalized populations, notably self-identifying Indigenous populations, are lost among these estimates. For example, the Indigenous Batwa of southwestern Uganda are an economically, politically, and socially marginalized population that resides in the southwestern highlands of Uganda. A previous survey conducted in the districts of Kabale and Kisoro in 2011 estimated that only half of the Batwa households owned a mosquito net and nets were obtained from a variety of sources, such as international aid agencies, the government, and church organizations [23]. Only two percent of nets were purchased.

Herein, this research is situated in the southwestern Ugandan District of Kanungu, among 10 formal Indigenous Batwa communities. The aim of this study was to estimate the levels of ownership and use of LLINs, and identify the socioeconomic determinants of LLIN retention within an Indigenous Batwa population after a targeted LLIN distribution event. Specific objectives included to: 1) estimate the pre- and post-distribution levels of ownership and within household access to LLINs; 2) describe individual-level LLIN use and non-use patterns at 3 and 18-months post-distribution; and 3) evaluate socioeconomic factors that affect household retention of LLINs post-distribution.

## Materials and Methods

### Study Population

The Batwa of Kanungu District, southwestern Uganda, are an Indigenous population traditionally based on forest hunter-gatherer livelihoods in what has now been established as the Bwindi Impenetrable National Park [24]. Due to recent environmental conservation, animal protection, and ecotourism activities in the park, the Batwa were forced to relocate to lowland areas and take up sedentary-agrarian livelihoods [25–27]. The Batwa continue to live in settlements characterized by poorer conditions with less access to adequate shelter, safe water, and sanitation infrastructure than their non-Indigenous neighbors [23,28,29]. The majority of Batwa adults are engaged in income generating activities, mostly working as laborers on agricultural fields, or casual laborers [23].

### Distribution of LLINs

A targeted distribution of long lasting insecticidal nets (LLIN) was undertaken in November 2012 by an international research team (the Indigenous Health Adptation to Climate Change (IHACC) group: <www.ihacc.ca>) working in collaboration with McGill University, the Ugandan Ministry of Health, Makerere University, and local development agencies, including the Batwa Development Programme (BDP). Two LLINs were provided free of charge to each Batwa household present at the time of the distribution in each of the 10 formal Batwa communities in Kanungu, which resulted in 98% of eligible households receiving two nets. The list of eligible households was derived from a full listing of households in the 10 Batwa settlements, maintained by a local community development organization. Two nets were given regardless of prior net ownership within the household. LLINs were 100% polyester and treated with deltamethrin (80mg/m^2^), effective with a median lifespan of approximately three years. Education on the proper usage of nets occurred at the time of distribution, and a local health worker visited the communities every few months to provide education on the causes of malaria and malaria prevention. A list of households that received the IHACC LLINs was linked to the subsequent surveys with unique household identifiers assigned by the IHACC research group.

Uganda had two universal coverage campaigns prior to and during the study period. However, the Batwa population only makes up 1% of the total population of residents in Kanungu District, and this marginalized population was not specifically targeted in these campaigns. Some examples of net distribution activities in Kanungu during the study period include: 1) in 2011, one of the major hospitals, as part of a regional initiative funded by the government of Uganda, distributed subsidized nets to pregnant mothers and children under five years old in the area at a cost of 3000 Ugandan shillings (~ $1.00 US); 2) in 2013, several thousand nets were donated to be distributed to young children (<5) and expectant mothers through the health centers in the area; and 3) in 2014, the Bwindi Community Hospital distributed 300 ITNs to the highest risk areas during malaria awareness week.

### Longitudinal Monitoring

**Fig 1:**
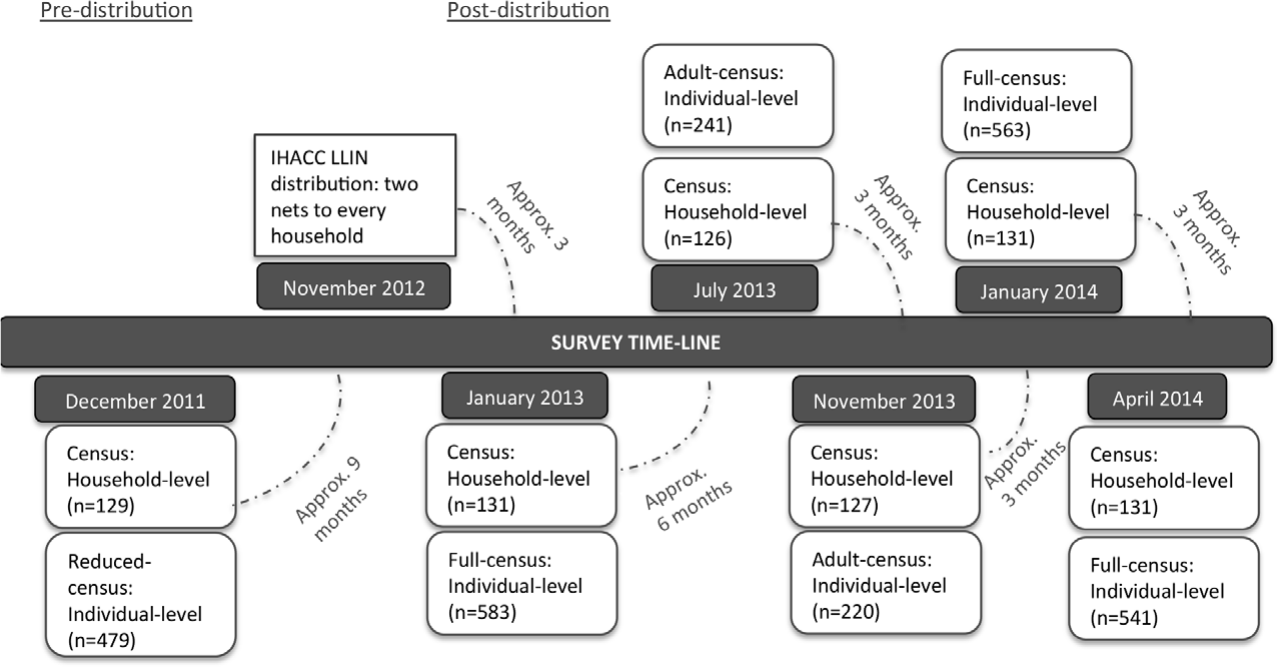
Schematic timeline of long lasting insecticidal net (LLIN) distribution and cross-sectional survey implementation from December 2011-April 2014 among Batwa in Kanungu District, southwestern Uganda.

In total, six cross-sectional, face-to-face, attempted census surveys were administered: one pre-distribution (December 2011), and five post-distribution at approximately 3-month intervals from January 2013-April 2014 (Fig 1). Each survey attempted to capture a census of the Batwa population in Kanungu District. Non-participation in a survey was either the result of a decline to participate (proportion of individuals that declined ranged from 1–4%) or where contact could not be made because the individual or household was away from the community. Three individual-level surveys captured Batwa of all ages and were conducted post-distribution: January 2013 (*n* = 583), January 2014 *(n* = 563), and April 2014 (*n* = 541). Individual-level surveys conducted in July 2013 and November 2013 captured a survey of adults only (≥18 yrs) and were not used for the *individual-level* analyses presented here. An attempted census of households was conducted in all six of the cross-sectional surveys: December 2011 (*n* = 129), January 2013 (*n* = 131), July 2013 (*n* = 126), November 2013 (*n* = 127), January 2014 (*n* = 131), April 2014 (*n* = 131). Every household was approached to be surveyed irrespective of whether the household was new to the study after the distribution of LLINs in November 2012. The sample of households used in the analysis of *LLIN retention* was based on the households that were present for, and received nets during, the November 2012 distribution event. Households that entered into the communities after this event or were not available during the distribution were omitted from the sample. Thus, the total number of unique households in the post-LLIN distribution sample was 136. From this sample, 121 households were surveyed in January 2013, 109 in July 2013, 103 in November 2013, 111 in January 2014, and 106 households at the end of the study period in April 2014. The sample size varied slightly in each survey depending on whether the household was present for that survey.

### Data Collection

Each consenting member of the household that was present for the survey answered an individual-level questionnaire. Parents or guardians answered questionnaires on behalf of children under 12 years old, while children 12–18 years answered questions with parental consent. One household-level questionnaire was administered to the head of the household over the age of 18. The questionnaires were administered by trained, local surveyors, in the local language (Rukiga). The number and composition of households in each community were verified by the respective village chairperson during each survey and household heads were asked to identify all of their co-habitants. Individual-level questionnaires captured data on age, sex, education, employment, community of residence, whether the person reported sleeping under a LLIN the previous night, and perceptions of malaria. The household-level questionnaire captured data on household mosquito net ownership (i.e. number and source of nets) and whether the net(s) was hanging the previous night, household assets, and household composition.

### Definitions

The main study outcome post-LLIN distribution was the proportion (%) of households that reported owning a LLIN for any given survey. The numerator is the number of households reporting owning a LLIN and the denominator is the total number of households. Inclusion was restricted to households defined within our study sample (households that received LLINs during the November 2012 distribution). The rate of retention of LLINs between surveys was calculated as the difference in the proportion of LLIN ownership between time i (_Ti_) and time j (_Tj_) divided by time i. The proportion of the population with access to a LLIN was calculated as per Koenker & Kilian 2014. This population-level access indicator was calculated as the theoretical number of people with access to a LLIN in a household divided by the total number of people in the household. The theoretical number of people with access to a LLIN in a household was the number of LLINs multiplied by a factor of 2.0 [30]. If there were more theoretical than actual people in a household, the actual number was applied. The person-to-net ratio was calculated as the number of people in the household divided by the number of LLINs in a household, among LLIN owning households [31,32]. LLIN use, measured at the individual level, was defined as the proportion of individuals reporting that they slept under a mosquito net the previous night for any given survey. A LLIN use:access ratio was calculated by dividing the proportion of the population that slept under a LLIN by the proportion that had access to one. This indicator reflects the proportion of the population using LLINs, among those that have access to one within their household [30]. The proportion (%) of community-level LLIN use post-distribution was defined as the number of people reporting to have slept under a LLIN the previous night within a community over the total number of people surveyed in that community. The Batwa population is predominantly subsistence based; household wealth (presumed to be time-variant) was thus generated from the correlations of household asset indicators (S1 File) using a polychoric Principle Component Analysis (PCA) for categorical data [33]. Using household assets as a proxy for wealth has been determined to be a good predictor of health in rural contexts [34], and has been used as a predictor in similar studies [5,8]. We generated tertiles of the PCA wealth score using natural breaks. Amidst existing socioeconomic gradients within these communities, the population is relatively impoverished and households were thus classified as either least poor, poor, or poorest. We further calculated equity ratios, comparing the proportion of LLIN ownership between the lowest defined wealth tertile (poorest) and highest defined wealth tertile (least poor) Batwa households for each survey post-distribution to see if the gap in ownership increased, decreased, or remained stable over time [5]. A decreasing equity ratio value represents an increase in inequities in LLIN ownership (poorest households [numerator]; least poor households [denominator]). From a ‘mid-point’ survey (January 2013; three months after distribution), we created a household female adult education variable and a household adult education variable consisting of the highest reported education of any female/ adult over the age of 16. Given the low number of observations reporting education beyond primary school, the variables were binary: no formal education versus some primary or higher education. Adult education and female adult education have sometimes been shown to be good predictors of household health and uptake of prevention measures [12,35,36]. Time-variant discrete variables indicating household size are based on the number of people in the household and the number of rooms in the home. Finally, a binary variable was created to capture a household’s infrastructural capacity to hang a net, based on whether it had ‘iron sheets’ or ‘grass thatched or banana fiber’ roofing.

### Statistical Analysis

Individual-level descriptive statistics on LLIN use and non-use were based on data collected from the *individual-level surveys*, while household-level descriptive statistics and regression modeling of retention of LLINs were estimated from the *household-level surveys*. Statistics on the existing household ownership of LLINs pre-distribution (December 2011) are presented alongside post-distribution results (January 2013-April 2014) to facilitate a comparison of the Batwa’s retention of nets with prior baseline conditions.

#### Household LLIN ownership and access

The proportion (%) of households owning at least one net from each household-level cross-sectional survey and the rate of retention of nets between surveys were calculated. Mosquito nets of any kind and nets treated with insecticide were included in pre-distribution estimates; post-distribution estimates included LLINs only. The proportion of people with access to a LLIN within their household, the person-to-net ratio (among households owning a LLIN) and the median number of nets owned were calculated for each household survey. Community-level coverage was calculated at the end of the study period (April 2014) to examine variability between the 10 Batwa communities.

#### LLIN individual use and non-use

Batwa from the individual-level surveys three and 18-months post-distribution were classified into one of four mutually exclusive categories of LLIN use and non-use [20]: 1) living in a household with no LLIN present (*non-use)*; 2) living in a household that owns but is not hanging an LLIN (*non-use)*; 3) living in household hanging an LLIN but not sleeping under it *(non-use)*; and 4) sleeping under an LLIN *(use)* [20].

#### Determinants of household LLIN retention

Post-distribution household-level surveys were analyzed as paneled data of repeated measures (January 2013-April 2014). The outcome was households reporting ownership of an IHACC-distributed LLIN, noting that reported acquisition of nets from other sources in post-distribution surveys was minimal (range: 8–13 non-IHACC LLINs per survey). Predictor variables for consideration were determined *a priori* from the literature [7,12], and included household wealth, household adult female education or household adult education, number of people in the household, number of rooms in the home, and the type of roofing material, while a factor variable representing the survey month/year was used to control for a time effect. First, a series of simple (i.e. one fixed effect) logistic regression models controlling for a household effect with a random intercept were used to explore potential unconditional associations between predictor variables and the outcome variable. Potential collinearity between predictor variables was assessed using a Spearman’s rank correlation coefficient with a cutoff of 70%. Given the likely violation of the assumption of model independence from repeated measures on the same households, as well as the possible community-level effects on the outcome [19], we used a random effects model with random intercepts to account for household and community effects, which assume exchangeable correlation structures [37,38]. The model was built using a manual backwards elimination process, and the best-fit model was determined by Bayesian Information Criterian (BIC) statistics of model comparison [39]. Models that (i) did not account for any community-level effect, and (ii) had community as a fixed effect variable, were run separately and assessed, but not found to improve model fit. Furthermore, examination of the distribution of the model’s residuals against the time variable (season [month/year]), and between residuals at different time points, did not display evidence of an autoregressive (non-exchangeable) correlation structure to the data. Two-way interactions among all predictors that were significant in the best-fit model were assessed by iteratively inserting interaction-terms into the model and looking for a change of 25% or greater in the covariates or any significant p-values <0.05. Model diagnostics were assessed graphically by visually examining the assumptions of normalcy for the residuals and assumptions of homogeneity of the Best-Linear Unbiased Predictors (BLUPs) of the random effects.

### Ethics

Participants provided verbal informed consent for participation in each survey of the study and parental/ guardian verbal informed consent was obtained for all individuals under the age of 18. For each survey, the trained surveyor described the study aims and goals to the participants, provided information on resources and contacts if participants had questions, reiterated to the participants that they could leave the study at any time, and that non-participation in the study would not inhibit their inclusion in any further community or research activities. Verbal consent was deemed the most appropriate given low levels of adult literacy in the communities. Data on consent was transferred to, and retained in, a secure database protecting the anonymity of participants with unique identifiers; the longitudinal nature of the study requires participant tracking of participation in multiple surveys. This study, and the consent procedure, was approved by the McGill University Research Ethics Board and is consistent with the Canadian Tri-Council’s Policies and requirements for the Ethical Conduct of Research Involving Human Subjects. Given the nature of our data, data are available upon request from interested researchers; contact <ihacc.geog@mcgill.ca> with inquiries.

## Results

Using the ‘mid-point’ January 2013 survey, and from available estimates [40], the Batwa in our study generally reflect the total Batwa population in Kanungu District; the study had slightly more males (48.5% compared to 47% in the total population) and is slightly younger (mean age 20 years compared to 23 years in total population) [40]. The majority (63%) of Batwa over the age of 16 had no formal education, with only 33% obtaining some primary education. Multiple responses allowed, 84% of Batwa adults (> = 18) said that malaria is caused by a mosquito bite, 63% of adults responded that dirty water can cause malaria, 12% responded that eating certain foods can cause malaria, 25% responded that being outdoors too long can cause malaria, and 6% that worms can cause malaria (Table 1).

**Table 1:** Demographics and perceptions of malaria in 10 Batwa communities in Kanungu District, southwestern Uganda taken at the “mid-point” (January 2013), three months after LLIN distribution*.

	Total (%)	95% Confidence interval	Average (standard deviation)
***Individual demographics n = 583***			
**Gender**	*n = 582* (100)		
Male	282 (48)	[44–52]	
Female	300 (52)	[49–55]	
**Age**	*n = 583* (100)		
0–5	147 (25)	[22–28]	
6–12	116 (20)	[17–23]	
13–34	212 (36)	[32–40]	
35+	108 (19)	[15–23]	
**Adult (**≥**16) education**	*n = 301* (100)		
No formal education	190 (63)	[58–68]	
Some primary	100 (33)	[28–38]	
Primary or beyond	11 (4)	[2–6]	
**Female adult (**≥**16) education**	*n = 174* (100)		
No formal education	122 (70)	[63–77]	
Some primary	45 (26)	[20–39]	
Primary or beyond	(4)	[2–6]	
***Household demographics n = 131***			
**Family size**			4.7 (2.3)
**Household Wealth**	*n* = 130 (100)		
Poorest	61 (47)	[38–56]	
Poor	34 (26)	[19–33]	
Least poor	35 (27)	[19–35]	
***Individual perceptions of malaria (adults (> = 18)***			
**How does one catch malaria?** **	*n* = 283 (100)		
Mosquito bite	238 (84)	[79–89]	
Dirty water	179 (63)	[57–69]	
Eating certain foods	34 (12)	[9–15]	
Catching a cold	12 (4)	[2–6]	
Being out in the sun too long	72 (25)	[21–29]	
Worms	16 (6)	[3–9]	

* Statistics from the attempted census of all Batwa who participated in the January 2013 survey

**Multiple responses allowed per individual. Each response is out of 100% if they answered yes or no

### Household ownership of, and access to, LLINs

Pre-distribution data show that, of the 129 households captured in the December 2011 survey, only 25% reported owning at least one mosquito net of any kind [95% CI 19–33]. Further, 19% of households reported that their net(s) had been treated with insecticide. For households reporting net ownership, the median number of nets owned was 1 [range: 1–5]. There was on average 5 people per net [CI 4.0–5.8] in Batwa households approximately a year before LLIN distribution.

**Fig 2:**
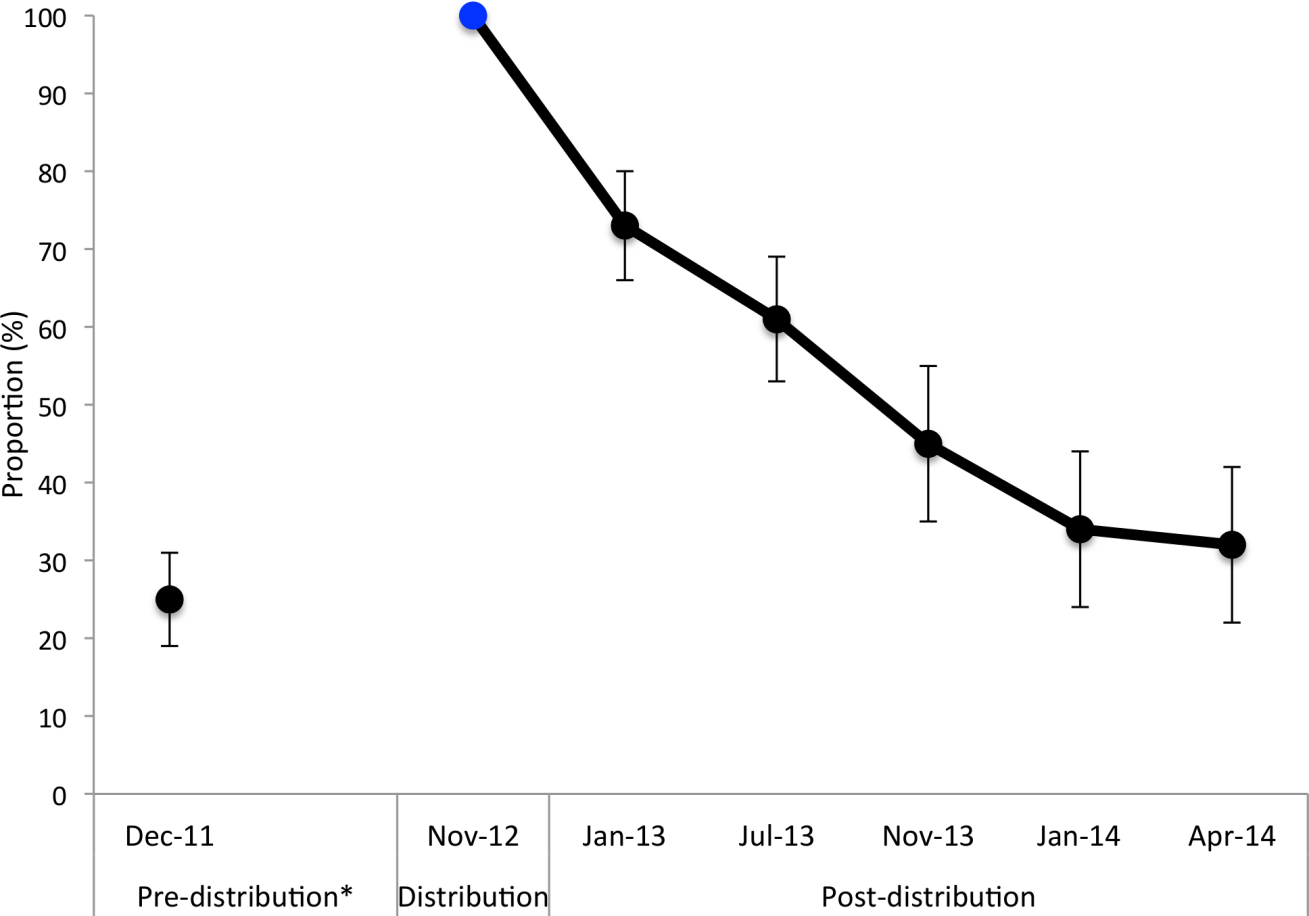
Proportion of households that reported owning at least one net pre and post-distribution in 10 Batwa communities in southwestern Uganda. Households owning at least one mosquito net of any kind was calculated for December 2011 and those owning at least one LLIN was calculated for the post-distribution estimates. Post-distribution estimates include LLINs obtained outside of the IHACC LLIN distribution, though acquisition of nets from other sources was minimal.

LLIN ownership decreased notably over the year and a half following LLIN distribution. In January 2013, three months after the LLIN distribution, only 73% [95% CI 64–80] of Batwa households reported owning a LLIN. In the following surveys, 61% [CI 51–69] of households in July 2013, 45% [CI 35–55] of households in November 2013, 34% [CI 26–44] of households in January 2014, and 32% [CI 24–42] of households in April 2014 reported owning a LLIN (Table 2, Fig 2). The greatest percentage change between surveys in the proportion of households owning a LLIN occurred in the first three months following distribution (27% decrease). Over the year and a half study period, there was an overall 68% decrease in households owning at least one LLIN.

**Table 2:** Household ownership, access, and use of LLINs 3 to 18-months post distribution among 10 Batwa communities (January 2013-April 2014).

Post-distribution	Number of households surveyed	Ownership: proportion of households owning at least one LLIN	Access: % of the population with access to a LLIN within their household	Use: Proportion of the population that slept under a LLIN *	Use:access: Ratio of use to access*	Person/net: Mean person to net ratio (among households owning a LLIN)	Median number of nets owned (all households, net owning households)
**3-months (January 2013; Dry season)**	N = 131	73% [64–80]	53% [46–60]	47% 43–51]	0.89	2.8 [2.5–3.3]	1, 2
**9-months (July 2013; Dry season)**	N = 126	61% [51–69]	43%[36–51]	-	-	3.3 [2.8–3.9]	0, 2
**12-months (November 2013; Mild rainy season)**	N = 127	45% [35–55]	33%[26–41]	-	-	3.0 [2.5–3.5]	0, 2
**15-months (January 2014; Dry season)**	N = 131	34% [26–44]	24%[17–30]	21%[18–25]	0.87	3.5 [2.9–4.3]	0, 2
**18-months (April 2014; Rainy season)**	N = 131	32% [24–42]	19%[14–25]	21%[18–25]	1.10	3.8 [3.1–4.5]	0, 1.5

* Use and use:access ratios were only calculated for January 2013, January 2014, and April 2014 when the full individual level surveys–used to calculate individual use of LLINs–were available.

Three months after the LLIN distribution (January 2013), 53% [95% CI 46–60] of the population had access to a LLIN within their own household. This proportion decreased steadily over the observation period with only 19% [95%CI 14–25] of the population having access to a LLIN 18-months post distribution. A similar trend was observed with net use: 47% [95% CI 43–51] of the population at 3-months post-distribution, and 21% [95% CI 18–25] of the population at 18-months post-distribution, reported sleeping under a LLIN the night before the survey. The ratio of LLIN use to access was 0.89 at 3-months, and 1.10 at 18-months, post distribution [30]. Among net owning households, the mean person-to-net ratio 3-months post-distribution was 2.8 [95% CI 2.5–3.3] people per net in a household. 18-months post distribution this number increased to an average of 3.8 [95% CI 3.1–4.5] people per net in a household. Among households owning at least one LLIN, there was a consistent median of 2 LLINs per household at each survey time point, except at 18-months post distribution when this median decreased to 1.5. (Table 2).

There was notable variation in household ownership and individual use of LLINs amongst the 10 Batwa communities (p<0.05) at the end of the study period (April 2014). Community-specific rates of household LLIN ownership varied, with two communities achieving >50%, while others had lower LLIN ownership rates of 20–50% (6 communities) and <20% (2 communities). Individual use of nets also varied considerably amongst communities. In half of the communities (n = 5), 20–35% of community members reported sleeping under a LLIN the previous night, while usage rates were <20% in the other five communities.

### Individual LLIN use and non-use

**Fig 3:**
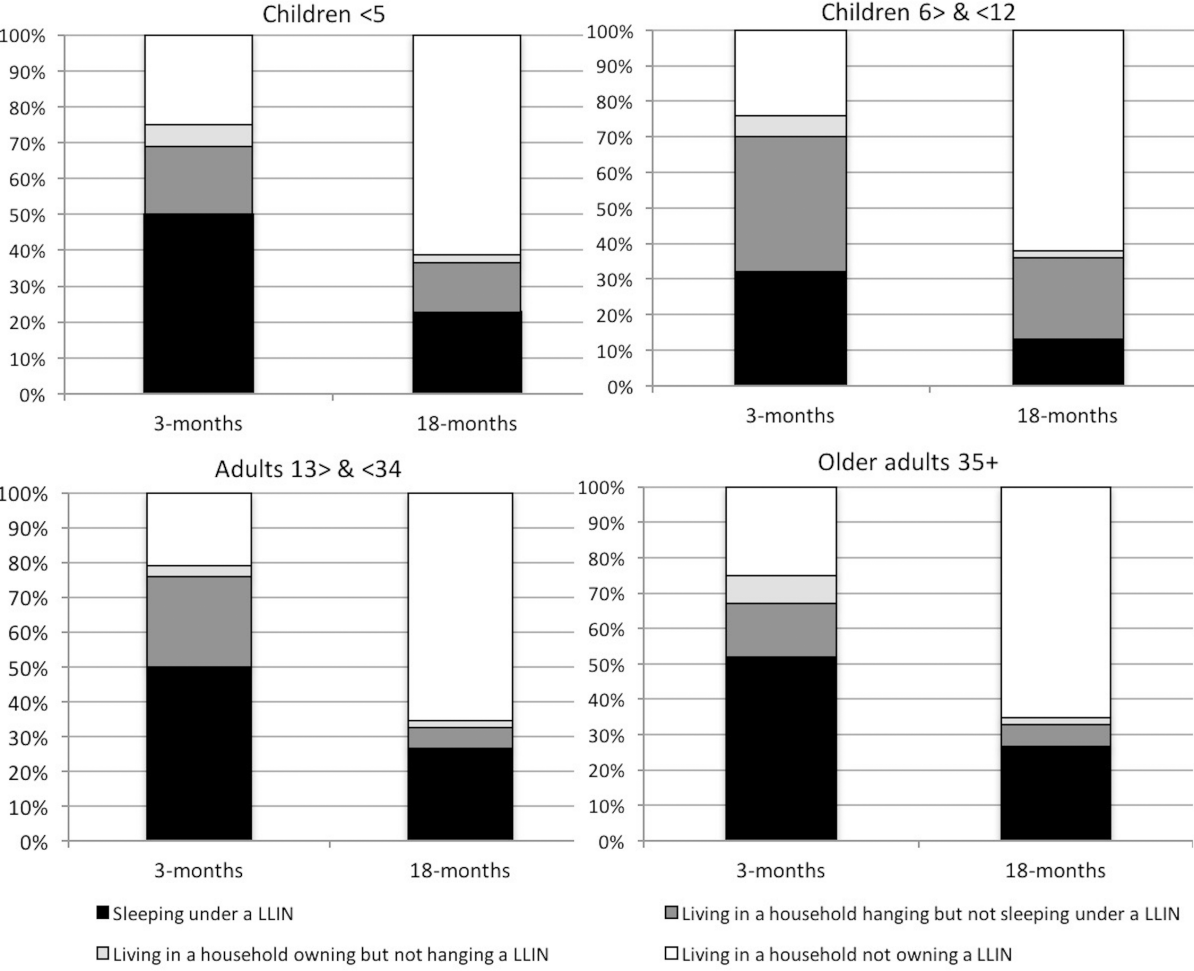
Proportion of Batwa by age group in net use and non-use categories 3- and 18-months post-distribution in 10 Batwa communities, Kanungu district, southwestern Uganda. Denominators include all Batwa in each age group within the study sample. *LLIN use category*: sleeping under an LLIN (black). *LLIN non-use categories*: living in households hanging but not sleeping under an LLIN (dark grey); living in households owning but not hanging an LLIN (light grey); living in household not owning an LLIN (white) [20].

Three- and 18-months following distribution, results indicate a rapid decline in LLIN use among the Batwa (Fig 3). As mentioned above, in January 2013, three months post-distribution, 47% of Batwa reported sleeping under a LLIN the previous night; 18-months later, this proportion declined to 21%. In April 2014, 18-months post-distribution, the majority of individuals who did not sleep under a LLIN lived in a household that did not own a LLIN (62%). For all age categories, the proportion of individuals living in households hanging nets but not sleeping under them was higher three-months post distribution compared to 18-months later. LLIN hanging was not a key constraint to LLIN use by individuals living in households that owned at least one LLIN and made the smallest contribution to non-use in every age group, reflecting less than 6% in any given survey.

Three months post-distribution, 50% of children under five years old reported sleeping under a LLIN the previous night. Under-five LLIN usage decreased to 20% at 18-months post-distribution, with 62% living in households that did not own a LLIN. The usage gap was most marked for children 6–12 years old across both time periods; this age group consistently comprised the largest proportion of individuals who lived in a household with a LLIN hanging, but who did not sleep under a LLIN the previous night (January 2013: 32%, April 2014: 23%). Three months post-distribution, half of adults 13–34 years were sleeping under a LLIN, but a year and a half later, 62% were living in households not owning a LLIN. Adults 35 years and older consistently comprised the greatest proportion of individuals who slept under a LLIN, relative to other age groups. No significant difference in LLIN usage by males and females was observed in any LLIN usage category. Details are provided in an Additional file (S2 File).

### Equity of LLIN retention

**Fig 4:**
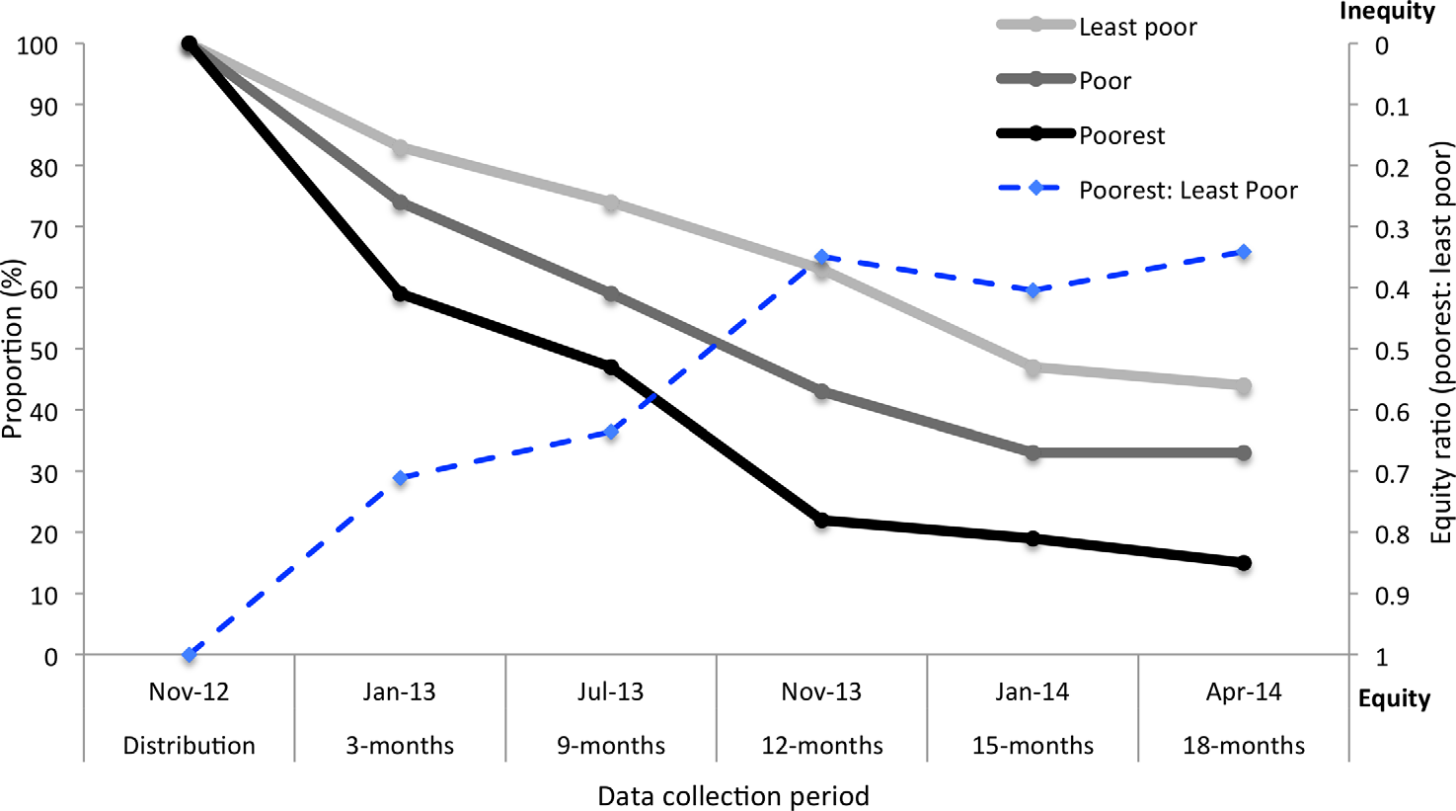
Proportion of Batwa households by wealth tertile reporting ownership of a LLIN post-distribution and the equity ratio in ownership between the poorest and least poor households in 10 Batwa communities in Kanungu, over a year and a half period. The Y-axis for the equity ratio is inverted with increasing inequity towards a value of zero (top) and decreasing inequity (equity) towards a value of one (bottom). The equity ratio was calculated with the poorest households as the numerator and the least poor households as the denominator.

Inequities in household retention of LLINs along socioeconomic tertiles were pervasive throughout the post-distribution study period. Consistent throughout each survey post-distribution, the least poor and poor Batwa households comprised a greater proportion of those owning a LLIN compared to the poorest Batwa households (Fig 4). The equity ratios of ownership (poorest: least poor wealth quintile) of IHACC-distributed nets steadily decreased over time, reflecting increasing inequities in retention of LLINs between socioeconomic groups. The ratio between the proportion of poorest and least poor households owning a LLIN 3-months post-distribution was 0.71 (poorest tertile = 59%: least poor tertile = 83%), at nine months it was 0.64 (poorest tertile = 47%: least poor tertile = 74%), at 12-months it was 0.35 (poorest tertile = 22%: least poor tertile = 63%), at 15-months it was 0.40 (poorest tertile = 19%: least poor tertile = 47%), and at 18-months it was 0.33 (poorest tertile = 15%: least poor tertile = 44%).

### Determinants of LLIN retention

LLIN retention in the model was further defined as self-reported ownership of at least one IHACC distributed LLIN. In the best-fit random effects multivariable logistic regression model, controlling for household and community-level effects, socioeconomic factors were significant predictors of households that retained at least one LLIN, keeping other included variables in the model constant (Table 3). The odds of owning at least one LLIN for the least poor households were 3.3 times greater [OR 3.3 95% CI 1.5–6.5] than for the poorest Batwa households (*p*<0.01). Highest adult female education in the household (*p* = 0.69), number of rooms in the household (*p* = 0.07), number of people (*p* = 0.38), and household roofing material (*p* = 0.16) were not significant predictors but improved the fit of the model and were retained as covariables. As expected, there was a significant global time effect (p<0.01) on the retention of LLINs in the model, with decreased odds of owning a LLIN with every subsequent survey from baseline. Model comparison using BIC values as the criterion indicated that controlling for household effects and including community as a higher level of clustering improved the model, even though the variance estimate between communities was lower than the variance within communities. The post estimation diagnostics indicated that the model was a good fit for the data.

**Table 3:** Best-fit model predicting household-level long lasting insecticidal net (LLIN) retention after a targeted distribution, controlling for repeated observations on households and community-level clustering among 10 Indigenous Batwa communities in Uganda.

Variable	Odds ratio	95% confidence interval	P-value
***Socioeconomic factors***			
**Household assets (tertiles)**			
Poorest	*Ref*	*Ref*	*Ref*
Poor	1.59	[0.79–2.77]	0.17
Least poor	3.32	[1.54–6.49]	<0.01
**Highest education of female ≥16 in household**			
No formal education	*Ref*	*Ref*	*Ref*
Some primary education and beyond	1.09	[0.70–1.68]	0.69
**Number of people in household**	1.05	[0.94–1.16]	0.38
**Number of rooms in home**	1.25	[0.98–1.62]	0.07
**Roofing material on home**			
Iron sheets	*Ref*	*Ref*	*Ref*
Grass thatched or banana fibers	0.34	[0.07–1.54]	0.16
***Time control***			
**Survey month/year**			0.01*
January 2013	*Ref*	*Ref*	*Ref*
July 2013	0.54	[0.27–1.08]	0.08
November 2013	0.22	[0.11–0.45]	<0.01
January 2014	0.17	[0.08–0.34]	<0.01
April 2014	0.12	[0.06–0.24]	<0.01

* Global significance of the variable in the model calculated from a Deviance test

## Discussion

Following a targeted, house-to-house distribution of LLINs in ten Indigenous Batwa communities in southwestern Uganda, Batwa households reported a decline in LLIN ownership over an 18-month period. The greatest reduction in net ownership occurred within the first three months following distribution, similar to Koenker et al (2014) in a multi-country study [13]. In our study, a year and a half after the LLIN distribution only 32% of households that initially received nets reported owning at least one LLIN. The observed rate of LLIN attrition for Batwa households six months after distribution, a year after distribution, and a year and a half after distribution, was among the highest found in comparable studies done in sub-Saharan Africa [5,6,8,9,11,13,14,16,41–43]. This highlights the need for better estimates of malaria intervention coverage for highly marginalized sub-populations in these regions. Similar to Larson et al (2014), in a study in Kenya, the median number of LLINs per household (among households owning LLINs) increased from 1 LLIN pre-distribution to 2 LLINs 3-months post-distribution, but these gains were not sustained at 18-months post-distribution [8]. Notably, 18-months post-distribution, there was an average of 3.8 people per LLIN in households owning a LLIN and only 19% of the Batwa population were estimated to be able to access a LLIN within their own household, which is far below the WHO’s global 80% target for universal coverage [30,32,43].

While Uganda has made notable gains in increasing overall ownership, access, and use of ITNs and LLINs over the past decade, our data suggest that the poorest and most marginalized populations, notably Indigenous populations, may still be disadvantaged from ownership and use of LLINs compared to the general Ugandan population [22]. An accurate assessment of sub-national disparities in ITN/LLIN ownership and population non-use is critical to inform malaria prevention programming and identify locally targeted entry points [20]. A year and a half after LLIN distribution in April 2014, the proportion of Batwa households that owned at least one LLIN was substantially lower than the estimated proportion of the Ugandan population that reported owning LLINs in the 2014 Uganda Malaria Indicator Survey (90% Uganda vs 32% Batwa) [22]. Furthermore, available estimates from 2013 among a small sample of neighboring non-Indigenous Bakiga in Kanungu District reported that 66% of Bakiga households reported owning at least one LLIN [21], almost double the Batwa’s household ownership at the end of our study period. Additional studies conducted in April 2014 among non-Indigenous Bakiga communities in Kanungu District found that 40% of all Bakiga study participants reported sleeping under a net the previous night [44], again almost twice the rate of LLIN usage by the Batwa at that time. These rates highlight the substantial disparity (and associated social gradients) in LLIN use and ownership between the Indigenous Batwa and their non-Indigenous neighbors, even following LLIN free-distribution efforts.

Just three months after the targeted Batwa LLIN distribution, which provided two LLINs to every household, less than half of the population reported sleeping under a LLIN the previous night; 18-months post LLIN distribution this proportion dropped as low as 21%. While overall use of LLINs decreased over the study period due to non-ownership, it is possible that the remaining nets were better used over time, consistent with our observation that the proportion of people living in a household hanging a net but themselves not sleeping under one decreased. However, this observed usage trend could also be explained by the seasonality of malaria transmission in the area, as the 3 and 18-months post distribution surveys were conducted during the dry and rainy seasons, respectively. For example, the 18-months post-distribution season was conducted in a period of heavy rains, likely resulting in higher mosquito densities, which has been shown to coincide with higher net hanging and usage rates [45]. Consistent over the study period, and with previous other studies, the highest proportion of LLIN use was among children under five and adults 35 years and older [8,46]. This may reflect the fact that, given biological vulnerability to malaria infection, children under 5 years and pregnant women are targeted globally for increased coverage and use of LLINs; consequently, information, education and behavior change messages are often tailored to promote net use for these groups [20]. In addition, parental-child sleeping arrangements have been found to reflect mosquito net use and may help to explain the observed trends. Studies in sub-Saharan Africa found that children who slept with their mothers were more likely to sleep under a net [47,48]. LLIN usage was disproportionately low for children 6–12 years old despite living in households that reported hanging a LLIN the previous night. Consistent with other studies in sub-Saharan Africa, this particular age group may be less prioritized for net usage, particularly when there are not enough nets to cover all members of the household [20,48–50]. Though in such cases, children may still receive some residual benefit from reduced vector abundance in households with LLINs hanging [2,51,52].

It is now well established that both global disease burden and access to preventive measures are patterned by socioeconomic gradients [53,54]. The association between wealth and willingness to pay for insecticidal nets is well recognized, with wealthier households more often acquiring nets in areas with market or subsidized access [7,55,56]. However, the relationship between wealth and retention of nets after a free LLIN distribution has been less studied, particularly using a longitudinal approach with paneled data [5,8,10,11]. Even though the Batwa in Kanungu appear to be a homogeneously poor population [57], within-population socioeconomic gradients do exist to pattern the retention of mosquito nets after a targeted distribution, similar to the findings of Hassan et al (2008) in Sudan [11]. Over time, the poorest Batwa households were less likely to retain a LLIN that was given out during the free distribution. Furthermore, the equity ratios of household LLIN ownership tended towards increasing inequity (ratio’s decreased) over the course of the study period, reflecting a widening of the gap in LLIN retention between the least poor and the poorest Batwa households. However, our findings are inconsistent with those of Larson et al (2014) that found, after a targeted LLIN distribution, social factors (i.e., maternal education and household wealth) no longer predicted the use of nets [8] and that inequities between social groups in net ownership and use were reduced [5–7,9]. The scale at which our study was conducted only allowed us to make statistical inferences about the impact of socioeconomic factors on retention of LLINs between Batwa households. However, given the relatively small socioeconomic gradients within the Batwa population [57] and the significant effect of household wealth on retention, we might expect to see an even greater relationship between social factors and retention of nets at the population-level, where social gradients between the Batwa and other non-Indigenous African populations are more pronounced [26,54,58].

While this study did not explore the reasons why nets were not kept, local informants working closely with the Batwa and past researchers in the field indicated that nets were often either sold/given away, used for other purposes, or discarded due to infrastructural limitations of thatched, semi-permanent housing [13,17,18]. Even though the LLINs were distributed with a clear message and demonstration of their purpose, and follow-up malaria prevention education occurred several times throughout the year post-distribution, household economic needs (i.e., selling nets or using for other purposes) may strongly influence LLIN retention for this marginalized population [8,17–19]. This presumption is further buttressed by our finding that the period with the greatest change in net ownership occurred within the first three months of the distribution–during a period of low malaria transmission (dry season)–suggesting sale or a loss or repurposing of nets before wear and tear from continual long-term use would likely occur [13,14]. A previous multi-country analysis found that nets obtained from a campaign were more likely to be given away—to family members, relatives, or others—compared to non-campaign nets [13]. For example, Koenker et al 2014 found that a large proportion (34%) of nets that were lost from households were given away and 63% of these were to relatives, particularly family members who reside elsewhere (i.e., students away at school) [13,59]. This study also found however that redistribution of nets was more likely to occur in net-rich environments where access to LLINs was high. Prior to the IHACC LLIN distribution, ownership and access to LLINs for Batwa was relatively low (<19% of households owned an insecticide treated net) suggesting it might be less likely that redistribution of nets between households would occur at a large scale within this population. However, redistribution to children at school is a possible mechanism of loss and warrants further investigation to optimize net retention among the Batwa. Past research from Nigeria found that net durability was associated with household socioeconomic status, with poorer households more likely to own nets in worse physical condition than less poor households. It is possible that this unmeasured variable (net durability) might have been a mediator on the pathway between household wealth and non-ownership of nets within our study [60]. In many East African Batwa communities, Batwa live in traditional grass thatched huts that may have inadequate infrastructure to properly hang LLINs [23,28]. However, within the context of our study, the majority of Batwa households were made of dried mud or brick and roofs were made of tin. From our analysis, only a small proportion of Batwa (<6%) reported living in a household that owned but did not hang a LLIN, and the lack of a statistically significant explanatory effect of roofing type and number of rooms on LLIN retention in the model further indicated that housing type was not a substantial driver of the sharp decline in LLIN ownership within this context.

This study has several limitations: first, our low sample size limited the power for statistical inference, even though our attempted-census surveys of the Batwa in Kanungu successfully covered a significant proportion of the total population. Conducting this research among a unique Indigenous population is both a strength and weakness of our study: it limits the generalizability of our results but offers critical insights into the situation of malaria prevention for a highly vulnerable population. Our study relies on self-reported measures, which could suffer from recall bias or be influenced by participant’s specific motivations or interpretation and understanding of a question [61]. It is possible that the Batwa under-reported LLIN ownership in the hope of receiving more nets, or that they over-reported LLIN ownership so as not to admit to their loss. However, it is likely that any reporting biases were consistent at each survey period and therefore the observed trends in LLIN retention and use over time are real. Pregnant women are defined as a biologically vulnerable group to malaria infection from reduced immunological protection and this study did not capture reliable data on pregnant Batwa women’s access to and use of LLINs. Given our findings, an in-depth qualitative inquiry into reasons for non-net ownership, perceptions of protective benefits, and seasonal adherence would be of value to provide an understanding of the effectiveness of targeted free LLIN distributions among marginalized populations.

## Conclusion

Direct house-to-house distribution of free LLINs did not result in sustainable increases in the ownership and use of LLINs among a population of Indigenous Batwa in Kanungu, Uganda. A year and a half after LLIN distribution, only one-third of Batwa households in Kanungu reported owning at least one LLIN, with levels of ownership reverting to pre-distribution estimates. Even after a targeted distribution, which aimed to reduce social, monetary and distance barriers in access to LLINs for these marginalized Indigenous communities, the use and ownership of LLINs among the Batwa remained below national and sub-national estimates, largely as a result of low LLIN retention. Within the Batwa communities, socioeconomic gradients were reinforced: the least poor Batwa households were significantly more likely to retain a LLIN compared to the poorest Batwa households. This research indicates that retention of freely distributed LLINs, particularly for impoverished populations, may remain subject to patterning by socioeconomic gradients over time. More effective longitudinal monitoring and evaluation programs are needed to assess the long-term impact of free LLIN distributions, and to identify contextually appropriate interventions to maximize the retention of nets, particularly for marginalized and vulnerable populations.

## Supporting Information

S1 File Relative wealth index: variable description and justification. (DOCX)

S2 File Classification of individual long lasting insecticidal net (LLIN) use and non-use for 10 Batwa communities in Kanungu District, Jan 2013 and April 2014, after a targeted free-distribution in November 2012 (% are calculated across rows within population sub-groups). (DOCX)

## Abbreviations

LLIN: Long lasting insecticidal net
ITN: Insecticide-treated net
IHACC: Indigenous Health Adaptation to Climate Change research group
BIC: Bayesian information criterion
HH: Household
