# Supplementary material for: A Longitudinal Analysis of Mosquito Net Ownership and Use in an Indigenous Batwa Population after a Targeted Distribution
Source: PLoS One. 2016 May 4;11(5):e0154808. doi: 10.1371/journal.pone.0154808 (PMC4856310; doi:10.1371/journal.pone.0154808)
Supplement: S1 File — (DOCX) [file pone.0154808.s001.docx]

**S1 File: Relative wealth index: variable description and justification**

The household wealth asset score was built using a polychoric Principle Components Analysis (PCA). The wealth score was built with a variety of variables that had the most statistical explanation of wealth but also contextually the most relevance. The Batwa are a special population that has in recent years had high exposure to foreign aid and development projects. Thus the researchers of this project, after initial qualitative investigation on indicators of wealth, chose variables based on a locally informed framework. This means that some variables that would be traditionally good indicators of asset based wealth (ie. roof material, flooring, latrine), for the Batwa do not indicate wealth, but instead indicate the presence of development projects in the area (i.e. development programs build houses for poorer Batwa). The variables that were selected as indicators of Batwa household wealth included: 1) use of soap, 2) owning animals, 3) owning a cellphone, 4) owning a radio, and 5) having family members away from the communities sending money home. The correlations of these variables explained 37% of the variation.
