# Supplementary material for: A Longitudinal Analysis of Mosquito Net Ownership and Use in an Indigenous Batwa Population after a Targeted Distribution
Source: PLoS One. 2016 May 4;11(5):e0154808. doi: 10.1371/journal.pone.0154808 (PMC4856310; doi:10.1371/journal.pone.0154808)
Supplement: S2 File — (DOCX) [file pone.0154808.s002.docx]

**S2 File. Classification of individual long lasting insecticidal net (LLIN) use and non-use for 10 Batwa communities in Kanungu District, Jan 2013 and April 2014, after a targeted free-distribution in November 2012** (% are calculated across rows within population sub-groups**).**

|  | Living in HH not owning LLIN (%) | Living in HH owning but not hanging LLIN (%) | Living in HH hanging LLIN but not sleeping under it (%) | Sleeping under LLIN (%) |
| --- | --- | --- | --- | --- |
| **January 2013** | | | | |
| **All** n= 576 | 126 (22) | 28 (5) | 151 (26) | 271 (47) |
| **Age**  **0-5** n= 147  **6-12** n= 116  **13-14** n= 207  **35+** n= 106 | 37 (25)  27 (24)  44 (21)  26 (25) | 8 (6)  7 (6)  6 (3)  9 (8) | 28 (19)  44 (32)  54 (26)  16 (15) | 74 (50)  38 (32)  103 (50)  55 (52) |
| **Gender**  **Male** n= 281  **Female** n= 295 | 64 (23)  62 (21) | 15 (5)  13 (4) | 69 (24)  83 (28) | 132 (47)  139 (47) |
| **April 2014** | | | | |
| **All** n= 541 | 341 (62) | 16 (3) | 77 (14) | 115 (21) |
| **Age**  **0-5** n= 131  **6-12** n= 114  **13-34** n= 179  **35+** n= 117 | 81 (62)  71 (62)  112 (62)  77 (66) | 3 (2)  2 (2)  8 (4)  3 (2) | 19 (14)  26 (23)  25 (13)  7 (6) | 30 (23)  15 (13)  40 (22)  30 (27) |
| **Gender**  **Male** n= 245  **Female** n= 287 | 152 (62)  182 (63) | 5 (2)  10 (3) | 36 (14)  41 (14) | 54 (22)  59 (21) |
